# Modeling of Efficient Control Strategies for LCC-HVDC Systems: A Case Study of Matiari–Lahore HVDC Power Transmission Line

**DOI:** 10.3390/s22072793

**Published:** 2022-04-06

**Authors:** Adeel Ahmed, Danish Khan, Ahmed Muddassir Khan, Muhammad Umair Mustafa, Manoj Kumar Panjwani, Muhammad Hanan, Ephraim Bonah Agyekum, Solomon Eghosa Uhunamure, Joshua Nosa Edokpayi

**Affiliations:** 1 School of Electrical and Electronics Engineering, North China Electric Power University, Beijing 102206, China; shahadeel041@gmail.com (A.A.); umairmustafa758@gmail.com (M.U.M.); mhanan@ncepu.edu.cn (M.H.); 2 Department of Electrical Engineering, Indus University, Karachi 75300, Pakistan; ahmed.muddassir@indus.edu.pk; 3 Department of Electrical Engineering, Sukkur IBA University, Sukkur 65200, Pakistan; manoj.panjwani@iba-suk.edu.pk; 4 Department of Nuclear and Renewable Energy, Ural Federal University, 620002 Ekaterinburg, Russia; 5 Faculty of Applied Sciences, Cape Peninsula University of Technology, P.O. Box 652, Cape Town 8000, South Africa; uhunamures@cput.ac.za; 6 Faculty of Science, Engineering and Agriculture, University of Venda, Private Bag X5050, Thohoyandou 0950, South Africa; joshua.edokpayi@univen.ac.za

**Keywords:** high voltage direct current (HVDC), line commutated converter (LCC), commutation failure, transient overvoltage (TOV), power transmission line

## Abstract

With the recent development in power electronic devices, HVDC (High Voltage Direct Current) systems have been recognized as the most prominent solution to transmit electric power economically. Today, several HVDC projects have been implemented physically. The conventional HVDC systems use grid commutation converters, and its commutation relies on an AC system for the provision of voltage. Due to this reason, there are possibilities of commutation failure during fault. Furthermore, once the DC (Direct Current) system power is interrupted momentarily, the reversal of work power is likely to cause transient over-voltage, which will endanger the safety of power grid operation. Hence, it is necessary to study the commutation failure and transient over-voltage issues. To tackle the above issues, in this paper, the dynamic and transient characteristics of Pakistan’s first HVDC project, i.e., the Matiari–Lahore ±660 kV transmission line has been analyzed in an electromagnetic transient model of PSCAD/EMTDC. Based on the characteristics of the DC and the off-angle after the failure, a new control strategy has been proposed. The HVDC system along with its proposed control strategy has been tested under various operating conditions. The proposed controller increases the speed of fault detection, reduces the drop of AC voltage and DC and suppresses the commutation failure probability of LCC-HVDC (line commutated converter- high voltage direct current).

## 1. Introduction

HVDC lines are being considered as the alternative to HVAC (high voltage alternating current) transmission lines due to low losses, cost-effectiveness on long distances, easy integration with renewable systems, and several other advantages [1,2]. Several countries including India, Pakistan, and China are shifting towards HVDC for new transmission lines installations. Pakistan has installed its first HVDC line named the Matiari–Lahore power transmission line ±660 kV [3].

Line Commutated Converter (LCC) based HVDC plays an important role in the transmission of power in bulk. China has had 20 LCC-HVDC operating since 1980 without any major fault [4]. Although LCC-based DC power systems have a large transmission capacity, they still need improvement by reducing the issues such as commutation failure, low black start capability, and high consumption of reactive power [5]. Among these problems, reducing the commutation failure provides a more reliable LCC-based HVDC line. The commutation failure involves the voltage commutation failure and transients over voltages (TOV). One of the major reasons behind the commutation problem is the significant requirement of reactive power at both sides of this HVDC system. This requirement originates due to the firing of thyristors after the commutation voltage becomes positive, which delays the current waveforms to the voltage waveforms [6].

To date, different types of solutions have been reported to overcome the risk of commutation failure through reactive power compensation [7,8], power-electronics-based strategies [9,10], and controller-modification-based strategies [11]. The power compensation and power-electronics-based strategies are not cost-effective and are also more complex. The comparison control systems modified strategies perform better without any disadvantages. There is a need to increase speed for fault detection to avoid major loss in the power system. AC faults near the inverter station reduce the AC voltage, as a result, the inverter reduces its extinction angle to maintain its AC voltage. Similar analyses on different LCC- HVDC power transmission lines have been reported by different researchers worldwide [3,4,12,13,14,15,16]. To overcome this issue, in this research work, an efficient control strategy has been proposed, which is faster than conventional control systems. The proposed control strategy improves the value of the extinction angle (*γ*) on the inverter side during fault conditions and improves fault detection speed. To analyze the performance of developed control strategies, the Matiari–Lahore HVDC transmission line has been simulated in PSCAD/EMTDC as a testbed. 

The transient state performance of the Matiari–Lahore LCC-HVDC system has been studied under single phase to ground fault and three-phase fault under different inverter’s SCR (Semi Control Rectifier) values. The analysis has been performed by suppressing the commutation failure and studying the TOV on the rectifier side, guaranteeing theoretical support in this specific LCC-HVDC system. Furthermore, a single control system has reported the suppression of commutation in terms of the AC voltage, DC, extinction angle, and firing angle. 

## 2. Modelling

**Figure 1:**
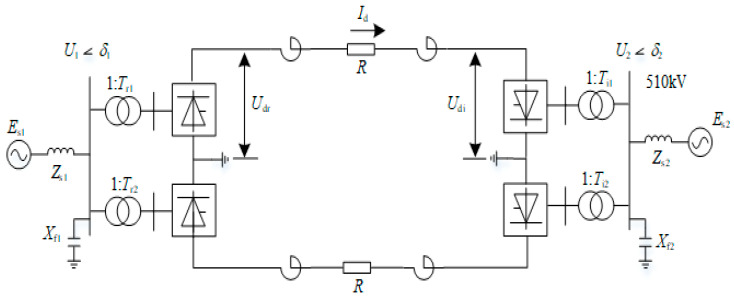
Circuit of LCC-HVDC system.

The basic structure of the LCC-HVDC is a ±660 kV/4000 MW, 12-pulse Bi-polar LCC-HVDC transmission line. The total rated power of the transmission link is 4000 MW, i.e., 2000 MW at each link on the rectifier and inverter side with an AC voltage of 510 kV at each link as shown in Figure 1.

$I_{d}$ is DC; $E_{s1}$ and $E_{s2}$ are an equivalent electromotive force in rectifier and inverter side; $Z_{s1}$ and $Z_{s2}$ are equivalent impedances of the AC system; $U_{1}$ is the voltage of AC bus connected to rectifier side; $T_{r1}$ and $T_{r2}\;$ are the ratio of converter transformers on the rectifier side; $U_{dr}$ and $U_{di}$ are DC voltages in rectifier and inverter side; R is the DC resistance of the line; *T_i_*_1_ and *T_i_*_2_ are the ratios of converter transformers on the inverter side; *U*_2_ is the voltage of bus bar on the inverter side; *X_f_*_1_ and *X_f_*_2_ are the reactance of AC filter in the rectifier and inverter side. The parameters of the LCC-HVDC system are shown in Table 1.

**Figure 2:**
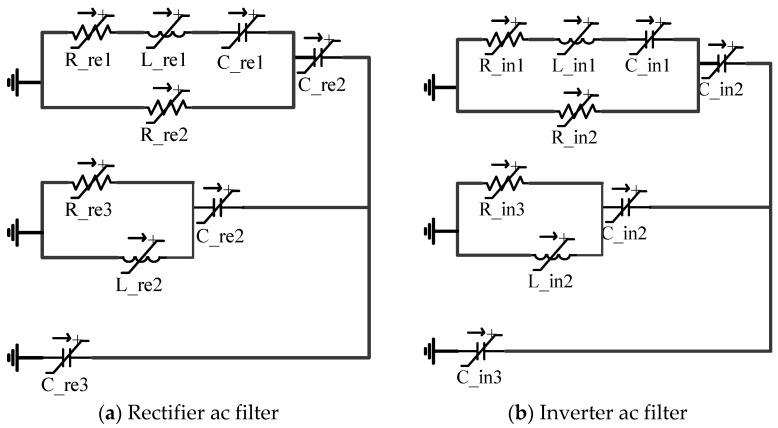
(**a**). AC filters installed at rectifier and (**b**) inverter side of the LCC-HVDC system.

It is well-known that where there are converters, there will be harmonic problems. Harmonics generated by converters are of the order of nP ± 1 on the AC side, where *P* is the number of pulses and n is an integer. AC filters are attached in parallel to the bus at both rectifier and inverter sides to avoid the AC system from high amplitude harmonics. The circuit diagram of these AC filters is shown in Figure 2a,b.

These filters also help in the compensation of reactive power. Parameters of the AC filter are provided in Table 2. 

Two sets of smoothing reactors, one at pole bus and one at neutral bus, are connected, each having a value of 150 mH. The size and numbers of these smoothing reactors are decided after considering several factors. In case of commutation failure at the inverter side, the DC peak value flowing through the converter valve and rate of current rise is limited to a specified limit and discharge current, which flows through the valve arrester will also be restricted under a specified limit. It will further avoid the resonance of the DC line at the fundamental and second harmonic frequency. The rated capacities of converter- and inverter-side transformers are 1207.46 MW and 1183.62 MVA, respectively, with positive sequence leakage reactance as 0.18 pu, and AC-side rated voltages are kept at 510 kV. All these values are adjusted by considering the typical LCC-based HVDC model and data of the Matiari–Lahore transmission line [15].

## 3. Mathematical Model of Converter Circuit

### 3.1. Valve Rating of Converter

The conversion from AC to DC and vice-versa is conducted in HVDC converter stations by using three-phase bridge converters. The configuration of the bridge (also called Graetz circuit) is a 6-pulse converter, and the 12-pulse converter is composed of two bridges in series supplied from 2 different (3-phase) transformers with voltages differing in phase by 30°. The average maximum DC voltage across the converter is given by [16]:

(1) $$V_{d0}=\frac{sq}{\pi }E_{m}\sin\frac{\pi }{q}$$

where “q” is the number of values, “r” the number of valves in parallel, and s is the number of valves in series in commutation group then:

p=(q)(r)(s)

The valve rating is specified in terms of Peak Inverse Voltage (*PIV*). For even *q*, the *PIV* valve is:

(2) $$PIV=2E_{m}$$

Moreover, when q is odd, then PIV has the following value:

(3) $$PIV=2E_{m}\cos\frac{\pi }{2q}$$

The ratio of PIV to average DC voltage is an index of valve utilization, which is given by Equation (4):

(4) $$\frac{PIV}{V_{d0}}=\frac{2\pi }{s\times q\times \sin\frac{\pi }{q}}\;(\text{For}\;q\;\text{Even})$$

(5) $$\frac{PIV}{V_{d0}}=\frac{\pi }{s\times q\times \sin\frac{\pi }{2q}}\;(\text{For}\;q\;\text{odd})$$

### 3.2. Converter Transformer Rating

The current rating of the valve is given by Equation (7) [17]:

(6) $$I_{v}=\frac{I_{d}}{r\sqrt{q}}$$

The transformed rating on the valve side is given by Equation (8):

(7) $$S_{tv}=p\frac{E_{m}}{\sqrt{2}}I_{v}$$

The transformer utilization factor (TUF) is given by Equation (8):

(8) $$TUF=\frac{S_{tv}}{V_{do}I_{d}}$$

### 3.3. Graetz Circuit without Overlap

At any instant, two valves are conducted in the bridge, one from the upper commutation group and the second from the lower commutation group. The instantaneous DC voltage $V_{d}$ during the interval is given by Equation (9):

(9) $$V_{d}=e_{b}-e_{c}=e_{bc}\;\mathrm{for}\;\alpha \leq \omega t\leq \alpha +60^{\circ }$$

(10) $$\mathrm{Let}\;e_{ba}=\sqrt{2}E_{LL}\sin\omega t\;$$

(11) $$\mathrm{then}\;e_{bc}=\sqrt{2}E_{LL}\sin(\omega t+60^{\circ })$$

(12) $$Average\;\mathrm{DC}\;\mathrm{Voltage}=V_{d}=\frac{3}{\pi }\int_{\alpha }^{\alpha +60^{\circ }}\sqrt{2}E_{LL}\sin(\omega t+60^{\circ })d\omega t=\frac{3}{\pi }\sqrt{2}E_{LL}(\cos(\alpha +60^{\circ }-\cos(\alpha +120^{\circ }))$$

(13) $$V_{d}=\frac{3\sqrt{2}}{\pi }E_{LL}\cos\alpha =1.35E_{LL}\cos\alpha$$

(14) $$V_{d}=V_{do}\cos\alpha$$

The above equations indicate that for different values of α, $V_{d}$ varies.

#### 3.3.1. DC Voltage Waveform

The rms value of the hth order harmonic in DC voltage is given by (15):

(15) $$V_{h}=V_{do}\frac{\sqrt{2}}{h^{2}-1}{\left(1+(h^{2}+1)\sin^{2}\alpha \right)}^{1/2}$$

#### 3.3.2. AC Current Waveform

The rms value of the fundamental component of current is given by (18):

(16) I1=122π∫−π3π3Idcosθ.dθ=6πId

The rms value of current is given by:

(17) $$I=\sqrt{\frac{2}{3}}I_{d}$$

The rms value of hth harmonics is given by

(18) $$I_{h}=\frac{I_{1}}{h}$$

#### 3.3.3. Power Factor

*AC* power supplied to converter is given by:

(19) $$P_{AC}=\sqrt{3}E_{LL}I_{1}\cos\phi$$

Neglecting the losses, *DC* power must be equal to *AC* power:

(20) $$P_{AC}=P_{DC}=V_{do}I_{d}=\sqrt{3}E_{LL}I_{1}\cos\phi$$

Inserting the value of $V_{do}$ from Equation (4) to Equation (20), we obtain:

(21) cosϕ=cosα

Equation (21) implies that the reactive power requirements will enhance when *α* will increase from 0, while only reactive power will be consumed if *α* is 90°.

## 4. Control System Design

**Figure 3:**
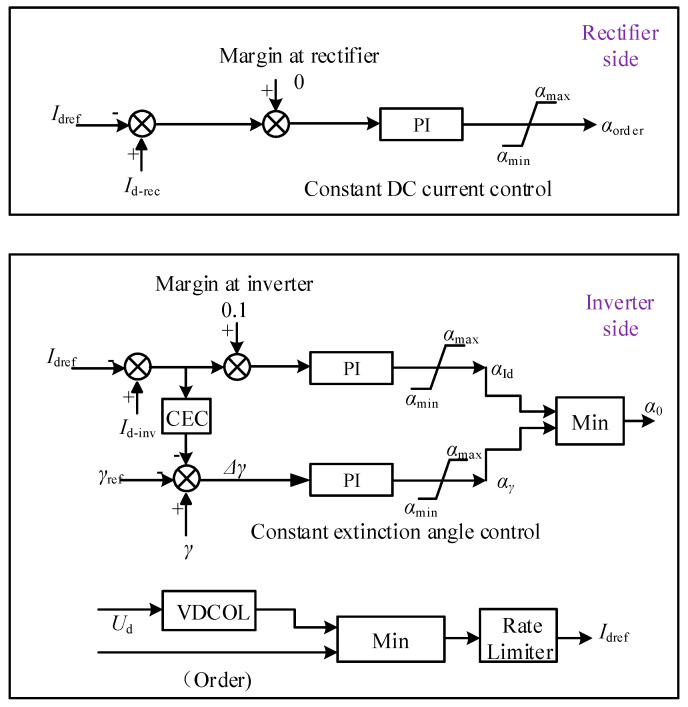
Control system of LCC-HVDC system.

The control system presented in Figure 3 operates in constant current control mode with a minimum firing angle at the rectifier side and adopts a constant extinction angle control (CEC) mode at inverter side. $I_{d\;ref}$ is the reference of DC; $I_{d-rec}$ is the measurement value of the DC, and *I_m_* is the margin current, which is zero on the rectifier side; $\alpha_{max}$ and $\alpha_{min}$ are the maximum and minimum limited value of the firing angle; $I_{d-inv}$ is the measurement value of DC on the inverter side; $\gamma_{dref}$ is the reference of the extinction angle on the inverter side; and *γ* is the measurement value of the extinction angle on the inverter side. The mathematical relationship for a constant DC voltage controller is given by:

(22) $$(I_{d-rec}-I_{dref}+Im)\;\left(K_{p}\;\times \;K_{i}+\frac{K_{i}}{s\;\times \;T_{i}\;}\right)=\alpha_{order}$$

The mathematical relationship for the constant excitation control is given by:

(23) $$(I_{d-inv}-I_{dref}+0.1)\;\left(K_{p}\;\times \;K_{i}+\frac{K_{i}}{s\;\times \;T_{i}\;}\right)\;=\alpha_{Id}$$

As *I_m_* = 0.1 for inverter side:

(24) $$\left(\Delta \gamma \right)\left(K_{p}\;\times \;K_{i}+\frac{K_{i}}{s\;\times \;T_{i}\;}\right)\;=\;\alpha_{\gamma }$$

The current margin at the inverter side is 0.1 pu, i.e., the voltage-dependent current order limiter (VDCOL) function is contained to avoid the DC overcurrent. 

## 5. Verification of Designed Model

The steady-state analysis of the LCC-HVDC model on the rectifier side is shown in Figure S1 provided in the Supplementary Materials. All values are reaching towards their steady-state as 1 pu (per unit), and α is reaching towards 20°. The rated DC voltage is 660 kV, the AC voltage is 510 kV, and the DC is 3 kA. The simulation results on steady-state analysis at the inverter side are shown in Figure S2 provided in the Supplementary Materials. All the values reach towards the steady-state at 1 pu, while the value of γ reaches towards its steady-state at 15°. The presentation of the steady-state values shown in Figures S1 and S2 indicates that the values are normal on both the inverter and rectifier sides. Figure S2 explains the results after the three-phase fault on the transmission side. It can be seen from Figure S2 that the AC voltages decrease from 1 pu to 0.96 pu, the DC voltages decrease from 1 pu to 0.70 pu, and the DC current increases from 1 pu to 1.07 pu, while the three-phase fault power decrease by 5% and *α* of rectifier side increases from 20° to 32° in transient state analysis. Similarly, changes have been noticed on the inverter side as shown in Figure S3 provided in the Supplementary Materials. 

It has been noticed from Figure S3 that the AC voltage decreases from 1 pu to 0.89 pu, the DC voltage also decreases from 1 pu to 0.82 pu, the DC increases from 1 pu to 1.07 pu, and the power of the inverter during the three-phase fault decreases by 1%. The extinction angle (*γ*) of the inverter side decreases from 15° to 9° during the transient state.

## 6. Transient over Voltage Evaluation

In this section, analysis was performed after obtaining TOV through the three values of SCR on the rectifier side, while on the inverter side, a fault will be applied. If the power is interrupted by blocking converter stations of the HVDC system and applying AC faults at inverter side converter stations leading to commutation failure, the reactive power of those RLC filters will flow into the AC system, which usually causes TOV [15,18]. Generally, a TOV of 30% is believed to be very high, and protection strategies are needed to avoid damage to the equipment. Generally, the TOV is lower with a lower impedance angle and vice versa [19]. 

**Figure 4:**
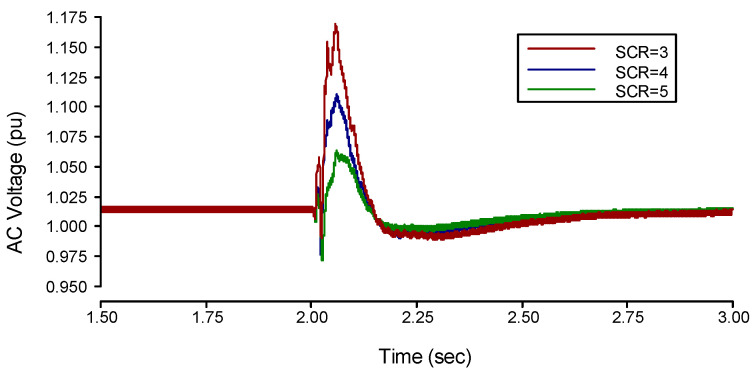
Transient State analysis of the LCC-HVDC system results on the Inverter side.

The three-phase fault on the inverter side is applied at three set values of SCR (3, 4, and 5). It has been noticed that the value of the TOV decreases with the increase in SCR values. It was 1.166 pu at 3, 1.08 pu at 4, and 1.061 pu at 5, as shown in the simulation results of Figure 4. 

The three-phase fault on the inverter side results in a rapid drop in AC voltage, and the AC system provides a more reactive power supply to the AC bus if the AC filter’s reactive power is in surplus, then this reactive power changes its flow from AC bus to the system.

**Figure 5:**
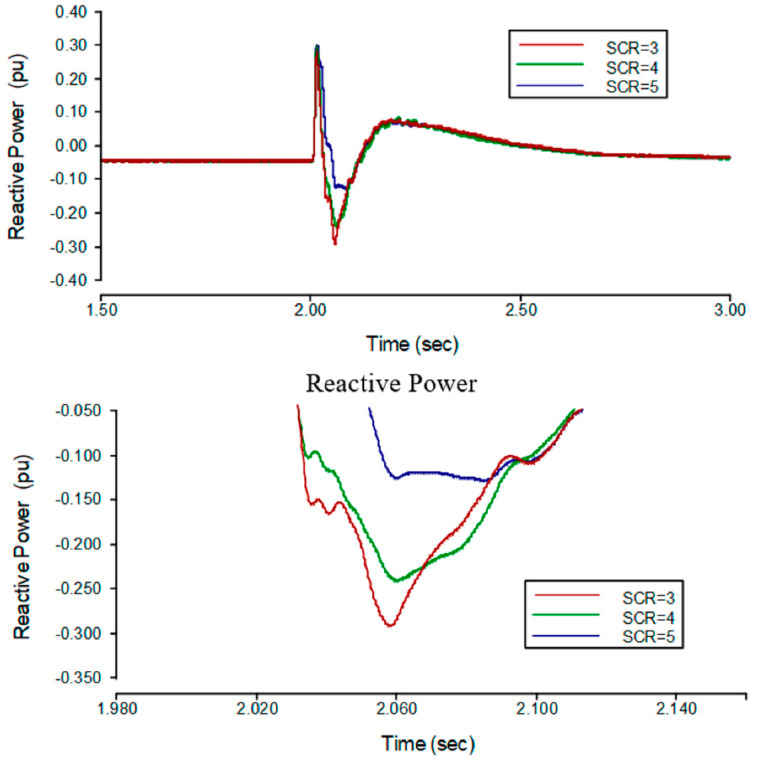
**Upper Graph** is the curves of reactive power on the rectifier side during transient state. **The lower graph** is the zoomed version of the upper graph that reveals the values clearer.

From Figure 5, when SCR is 3 the reactive power peak is −0.292 pu and the *TOV* of the rectifier side is 1.166 pu. When the SCR is 4 the reactive power peak is −0.241 pu and the *TOV* of the rectifier side decreases to 1.108 pu. When the SCR of the rectifier side increases to 5, the reactive power peak is −0.126 pu and the *TOV* will decrease to 1.061 pu. So, the *TOV* is related to the amount of reactive power flowing from the AC bus to the AC system. Hence, the fault applied at the AC bus on the inverter side leads towards the commutation failure. Furthermore, it will interrupt the power, and the reactive power consumption of the rectifier will be eliminated, which causes the surplus reactive power generation by the filters. This power will flow into the AC system and causes *TOV* at the rectifier side’s AC bus. Finally, if SCR is increased, it is beneficial to suppress surplus reactive power to flow into the AC system, hence, *TOV* can be reduced.

When one side of the system stops working, it is referred to as a monopolar block. In a monopolar block, the following simulation values are obtained against different SCR values. A declining trend in TOV has been reported, as is shown in Table 3.

When the whole system stops working, one can refer to it as a bipolar block. The same trend has been seen in a bipolar block. The results are shown in Table 4 and the curves of both sides are shown in Figure 5.

## 7. Commutation Failure Analysis

In this part, the transient state performance of the LCC-HVDC system is discussed for a single phase to ground fault and the three-phase fault under the variation of inverter’s SCR. The relationship of the DC and *γ* with commutation failure is explained, and a suppression method for commutation failure is proposed.

### 7.1. Transient State Performance in Single Phase to Ground Fault

A fault with the duration of 0.05 s is applied at 2.5 s, and the AC voltage drops from 5.6% to 0.959 pu at an SCR value of 3. The AC power of the rectifier side drops by 59.1% to 0.409 pu, and the *α* of the rectifier side increases up to 100°, as shown in Figure S5 in the supporting information. 

When the SCR of the inverter side changes to 5, the AC voltage drops by 3.2% to 0.983 pu, the AC power of the rectifier side drops by 6.5% to 0.935 pu, and the *α* of the rectifier side increases to 27°. 

On the inverter side, at SCR 3, the AC voltage drops by 11% to 0.889 pu, the AC power drops by 61% to 0.381 pu, the *γ* decreases to 0°, and the DC increases to 1.441 pu. On the SCR value of 5, the AC voltage drops by 2.4% to 0.976 pu, the AC power of the inverter side drops by 12% to 0.871 pu, the *γ* of the inverter side was 13°, and the DC increases up to 1.205 pu, as shown in Figure S6 of supporting information.

Concisely, the increment in SCR reduced the drop in AC voltage and AC power, further resulting in the increment of the γ; hence, it is beneficial to defend against the commutation failure of the LCC-HVDC system.

### 7.2. Transient State Performance in Three-Phase to Ground Fault

In a simulation, the three-phase fault with a duration of 0.05 s at the time of 2.5 s is applied on the inverter side’s AC bus along with an inductance of 1.33 H. On the rectifier side, at SCR 3, the AC voltage dropped by 2.3% to 0.977 pu, the AC power dropped by 81.9% to 0.181 pu, and the DC voltage of the rectifier side is dropped by 44.7% to 0.553 pu while the α of the rectifier side during the three-phase fault is increased to 90°. At SCR 5, the AC voltage dropped by 0.5% to 0.995 pu, the AC power dropped by 6.6% to 0.934 pu, the DC voltage dropped by 13.3% to 0.867 pu, and the α of rectifier side increased to 25°. The results for SCR 3 and 5 of the rectifier side are shown in supporting Figure S7 in the Supplementary Materials.

On the inverter side under the same fault conditions and SCR 3, the AC voltage dropped by 10.5% to 0.895 pu, the AC power dropped by 85.6% to 0.144 pu, the DC voltage of the inverter side dropped by 92.3% to 0.077 pu, the γ of the inverter decreased to 0°, and the DC increased to 1.236 pu. After a change in the SCR value from 3 to 5, the AC voltage drops by 2.7% to 0.973 pu, the AC power drops by 11% to 0.890 pu, the DC voltage drops by 11.7% to 0.883 pu, the *γ* of inverter side drops from 15° to 12°, and the DC increases to 1.031 pu, as shown in Figure S8 (in the Supplementary Materials). It is noticed that when the SCR value decreases then the drop in the AC voltage, AC power, and DC voltage have been reduced while *γ* is promoted. 

## 8. Suppression of Commutation Failure

**Figure 6:**
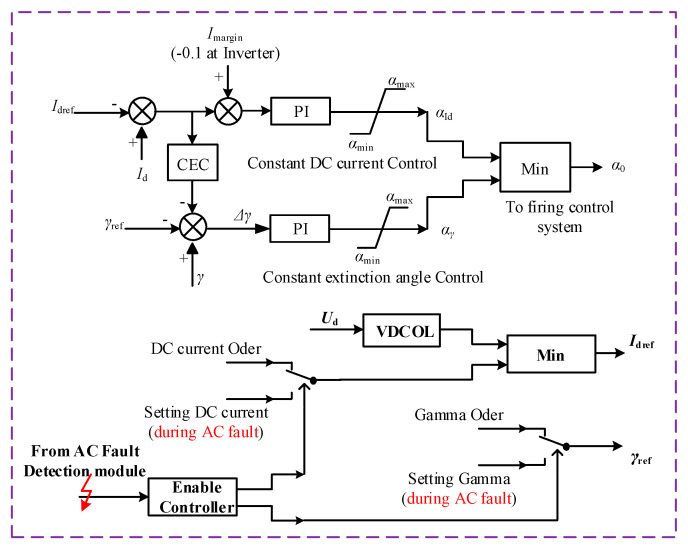
Proposed Control System.

In this section, a proposed suppression method to handle commutation failure under light AC fault and severe AC fault. The improved control system is designed to produce more precise reference values of DC and gamma for the inputs of constant DC and constant extinction angle. Furthermore, the Enable controller is added, which is missing in Figure 3’s system. The addition of the Enable controller reads the error signal in case of a fault and produces the signal accordingly to mitigate the fault. It can be seen from Figure 6 that the output of the Enable controller is fed as the inputs of Id reference and gamma reference. The verification of a proposed method has also been performed in this section through simulation in PSCAD/EMTDC. 

A valve that exists from a conducting state to the next specific valve continues to conduct. If the commutation process is not completed or the blocking capacity is not restored under the action of the reverse voltage, then the valve that was originally scheduled to exit will turn on again, and when the valve side voltage becomes positive, then the commutation failure occurs. It is believed that the inverters fail commutation only when the shut-off angle *γ* of the valve operation is less than its inherent limit shut-off angle $\gamma_{min}$ [19], where the thyristor $\gamma_{min}$ is a carrier composite switch in the thyristor element to establish a PN junction barrier layer to the time required to restore the positive blocking capability is usually about 10° [17]. The turn off angle can be expressed as:

(25) $$\gamma =\arccos(\frac{\sqrt{2}kX_{c}I_{\mathrm{dc}}}{U_{L}}+\cos\beta )$$

where *γ* is the turn-off angle, *β* is trigger lead angle, *k* is the converter transformer ratio, $I_{d}$ is DC, $X_{c}$ is equivalent commutation reactance, and $U_{L}$ is the RMS value of the AC bus voltage of the inverter. In case of asymmetrical fault, the relation will be:

(26) $$\gamma =\arccos(\frac{\sqrt{2}kX_{c}I_{\mathrm{dc}}}{U_{L}}+\cos\beta )-\phi$$

where *φ* is the phase aberration of the voltage waveform. The above mathematical equations explain the relationship between the DC and *γ*. Our proposed method is based on the above two equations. The CIGRE benchmark model of our proposed system is shown in Figure 6.

At the time of AC fault, the DC ordered value suddenly decreased from the rated value to a settled value of 0.55. Simultaneously, *γ*’s ordered value increased from 15° to 18°. Due to the delay in detecting an AC fault, the proposed measures will be applied after two circles of the time when AC faults occur. To verify our proposed method, we applied fault in two cases. Case 1 is without our proposed method and Case 2 is with the proposed method. Simulation has been performed for both cases with a single light and severe fault, as well as the three-phase light and severe faults.

### 8.1. Single Phase to Ground Fault

**Figure 7:**
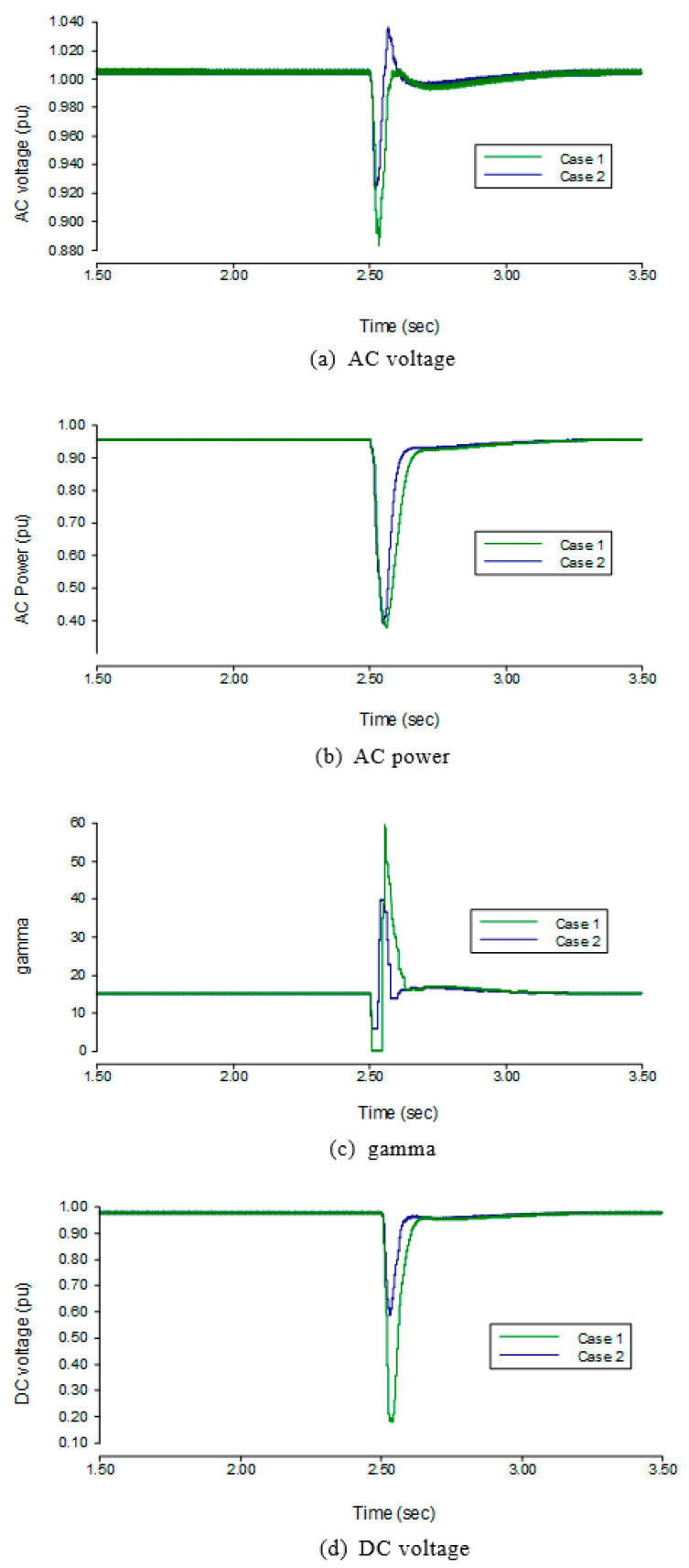

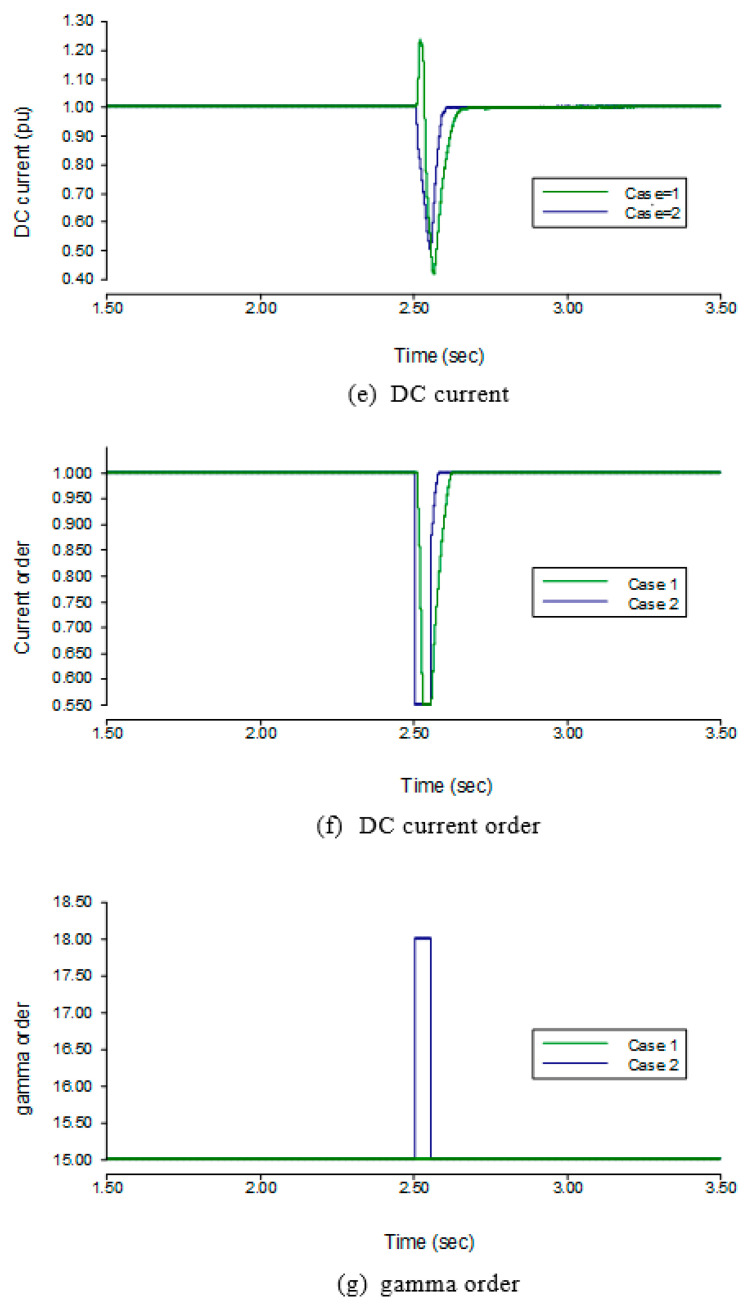
Transient state performance under single-phase to the ground with light AC fault.

The comparison results of the transient analysis are shown in the case of the application of light fault are shown in Figure 7. When the AC fault occurs at 2.5 s at the inverter side, the DC order in case 1 decreases at 2.512 s and drops from its rated value to 0.55 pu in 0.02 s. The DC order in case 2 shifted from its rated value to 0.55 pu sharply at 2.504 s, so the DC order in case 2 is arrives at a lower current value faster than in case 1. In addition, the *γ* order in case 2 increases from 15° to 18° at 2.504 s, whereas the *γ* order in case 1 always keeps its rated value. The results showed that the measured DC in case 1 increased to 1.236 pu much higher than the rated value when the measured DC in case 2 decreased. The measured γ in case 1 was 0°, while the measured γ in case 2 was of higher value 5°. Moreover, the sharp decrease in the DC order and increase in the γ order in the proposed control was useful to reduce the commutation failure probability of the LCC-HVDC. At the same time, the fluctuation of the DC voltage, the AC voltage and AC power in case 2 is less than that in case 1. When compared to the parameters in case 1, the drop in AC voltage at the inverter side is reduced by 0.051 pu in case 2, and the AC power of the inverter side drops by 0.02 pu. The DC voltage of the inverter side is dropped by 0.413 pu. Hence, the proposed method is beneficial to defend against commutation failure of LCC-HVDC system. 

In the case of a single phase severe fault, the obtained results’ behavior is similar, and it is provided in the Supplementary Materials in Figure S9. 

### 8.2. Three-Phase Fault

**Figure 8:**
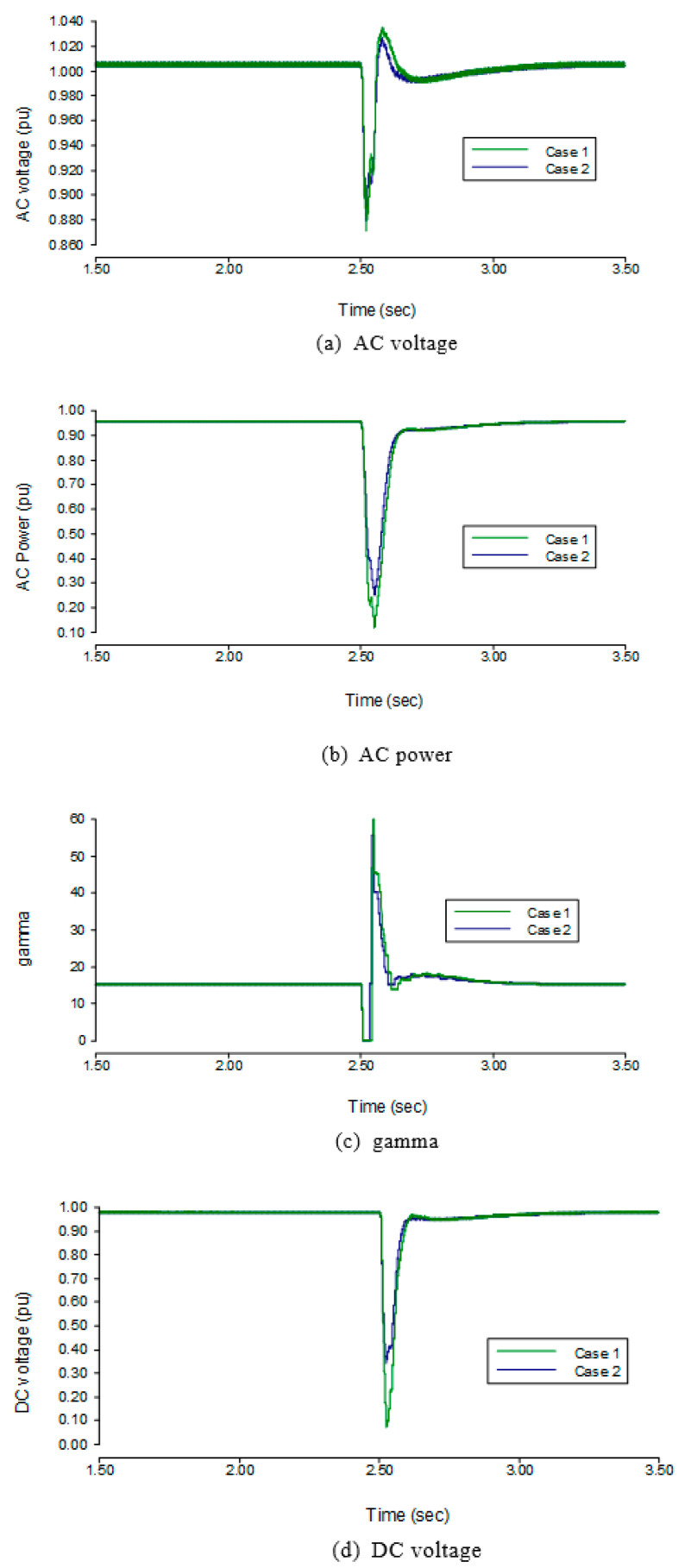

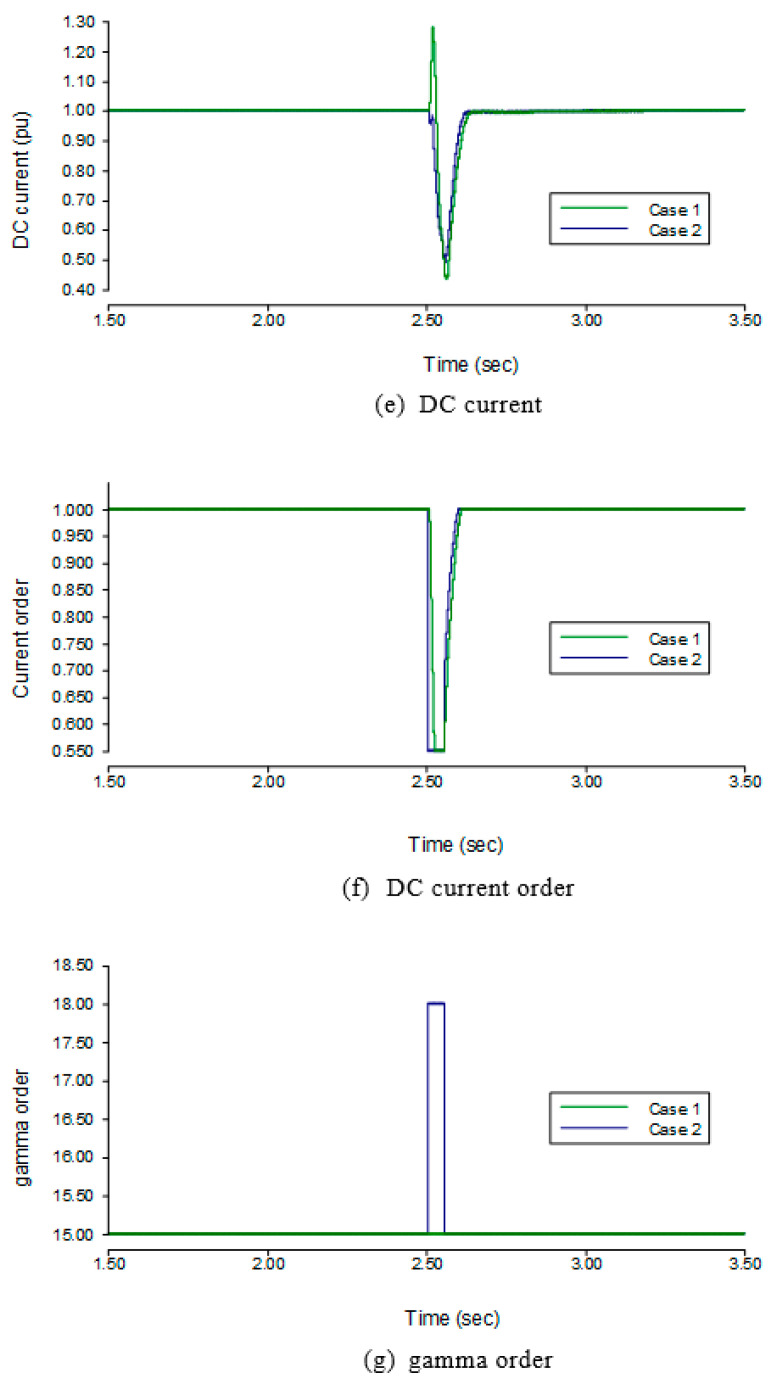
Transient state performance of three phase to ground with light AC fault.

The three-phase fault was applied on the inverter side’s AC bus along with an inductance of 1.33 H. The results on the rectifier side after the application of fault are shown in Figure 8. At an SCR value of 3, the AC voltage of the rectifier side drops by 2.3% to 0.977 pu, the AC power of the rectifier side drops by 81.9% to 0.181 pu, the DC voltage of the rectifier side drops by 44.7% to 0.553 pu, and the α of the rectifier side during the three-phase fault is increased to 90°. When the SCR of the inverter side is changed to 5, the AC voltage drops by 0.5% to 0.995 pu, the AC power of the rectifier side drops by 6.6% to 0.934 pu, the DC voltage of the rectifier side drops by 13.3% to 0.867 pu, and the α of rectifier side increases to 25°. It can be seen from the results that the SCR increases the drop in the AC voltage, AC power, and DC voltage. 

**Figure 9:**
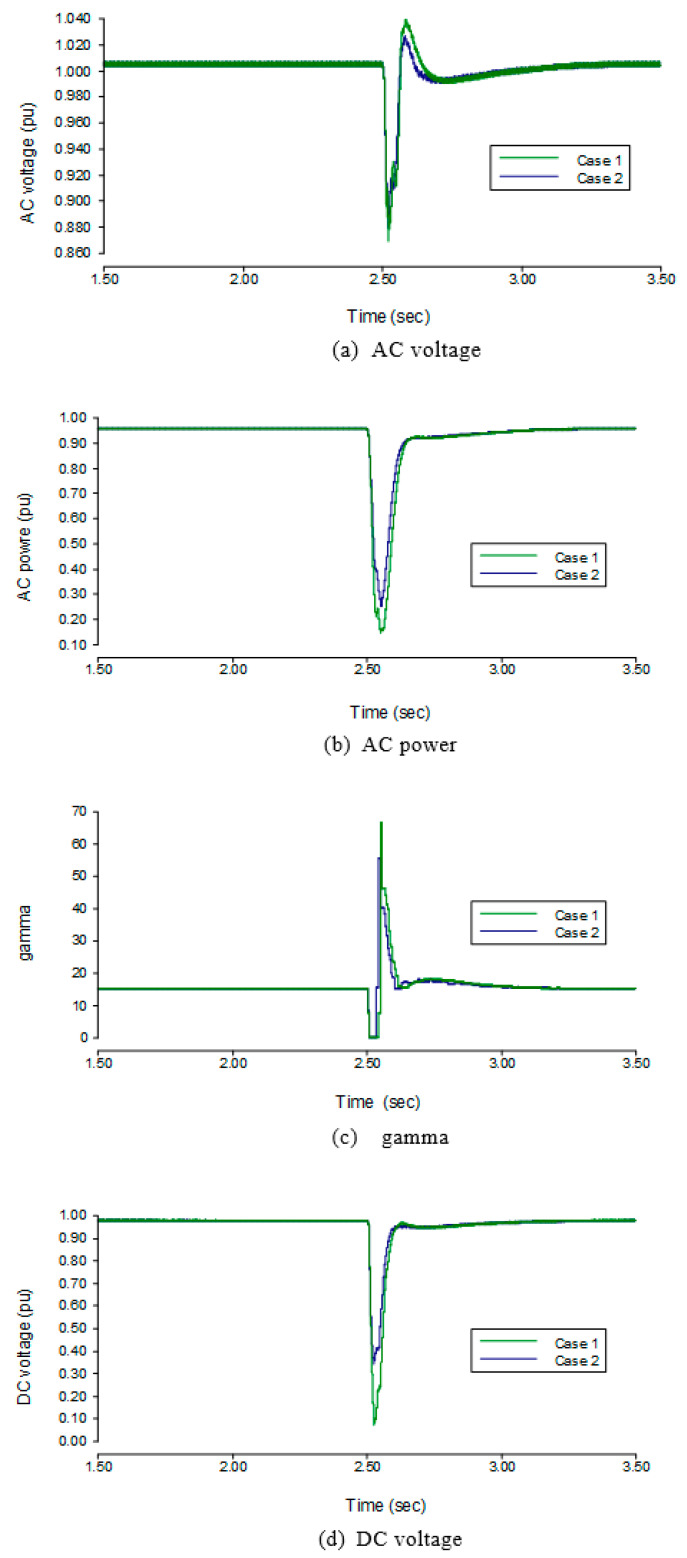

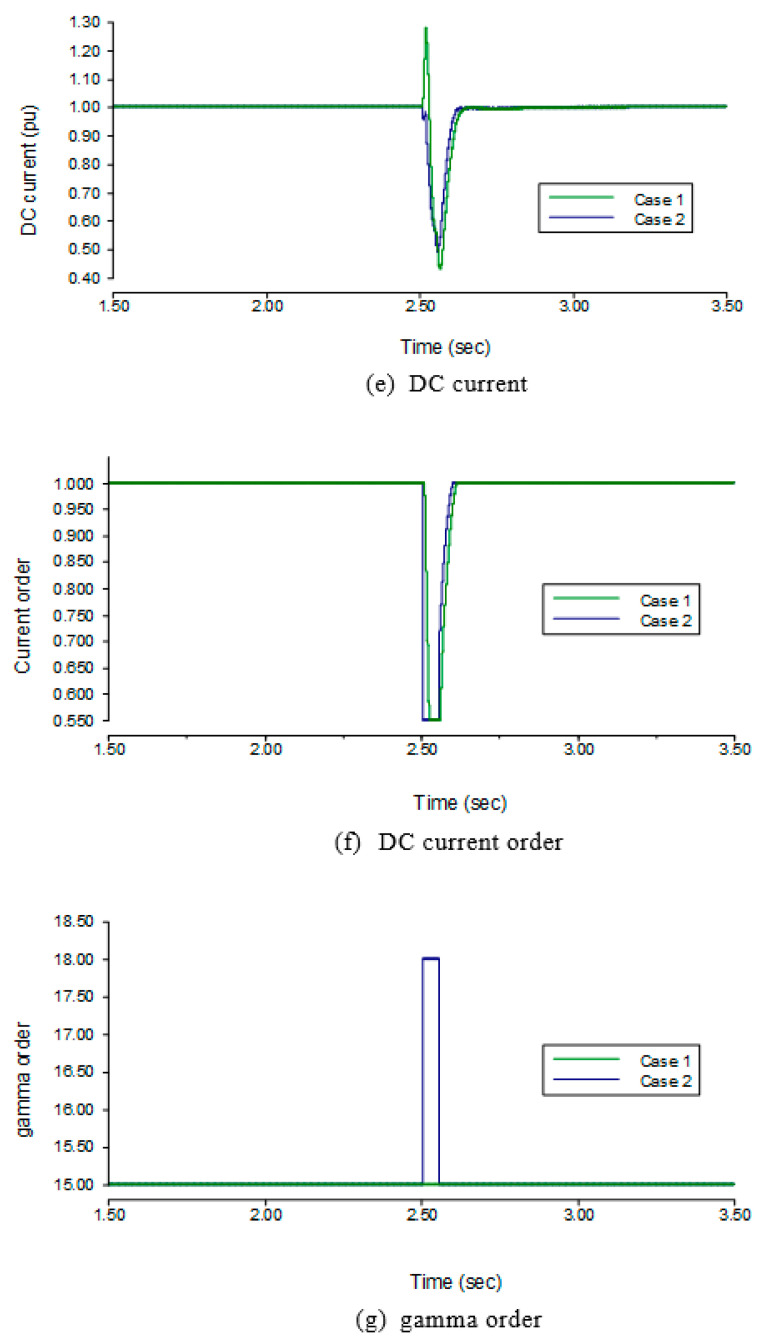
Transient state performance of the three-phase fault to the ground with severe AC fault.

The results on the inverter side in the three-phase fault are shown in Figure 9. While keeping the SCR at 3, a fault is applied at 2.5 s, and it can be seen from the results of Figure 8 that the AC voltage of the inverter side drops by 10.5% to 0.895 pu, the AC power of the inverter side drops by 85.6% to 0.144 pu, the DC voltage of the inverter side drops by 92.3% to 0.077 pu, and the γ of the inverter decrease up to 0° while the DC increases up to 1.236 pu. When the SCR is changed to 5, the AC voltage drops by 2.7% to 0.973 pu, the AC power of the inverter side drops by 11% to 0.890 pu, the DC voltage drops by 11.7% to 0.883 pu, the γ of inverter side drops from 15° to 12°, and the DC increases to 1.031 pu. It can be seen from the figures that when SCR increases, the drop in the AC voltage, AC power, and DC voltage are reduced and the *γ* is promoted during fault.

## 9. Conclusions

The equivalent model of the LCC-HVDC system is developed in PSCAD/EMTDC, and the control system of a model with all parameters and configurations is explained. After applying the steady-state and transient state analysis on the rectifier and inverter sides, the efficiency of the model is verified, and the obtained result indicated that the model could reach a rated value and meet the requirement for further study. In the simulation, an AC fault is applied on the inverter side, and the transient state performance on the rectifier side is studied. The TOV caused by monopolar and bipolar blocks is studied in detail under different SCR values. It is concluded that the TOV under a bipolar block is higher than that under a monopolar block. When SCR increases, the TOV is suppressed greatly. In the transient state performance of the LCC-HVDC system under single phase to ground fault and the three-phase fault, the mechanism of commutation failure of the LCC-HVDC is analyzed. It is found that the rapid rise of the DC and γ are closely related to the commutation failure of the LCC-HVDC, then the suppression method of commutation failure is proposed. The efficiency of the proposed suppression method is verified by simulation under a single phase to ground fault and the three-phase fault with light AC fault and severe AC fault. It is concluded that the proposed method is beneficial to defend against commutation failure of the LCC-HVDC system under a single phase to ground fault. The proposed method has little improvement in suppressing the α’s drop under the three-phase fault because when the fault is serious enough, the fast evolution of transient DC and AC voltage will limit the functionality of the proposed method. The thoughts, procedures, and indices established in this paper have great potential for further research in the area of the LCC-HVDC system. Commutation failure leads to the interruption of power, and it is harmful to the entire electrical power system. To avoid commutation failure and give better protection, the identification of the fault is very important, and it is necessary to detect, classify, and clear the faults as fast as possible. It needs further study on the fast way to detect the fault in the LCC-HVDC system and decide when to apply the suppression method of commutation failure. It also needs further study on the quantitative evaluation index to evaluate the commutation of the LCC-HVDC system and evaluate the effect of the proposed method of suppressing the commutation failure.

## Figures and Tables

**Table 1:** Parameters of LCC-HVDC model.

Item	Rectifier Side	Inverter Side
DC Voltage/kV	±660	±660
AC Voltage RMS/kV	510	510
DC Current/kA	3.03	3.03
SCR	3	3
Gain (I)	0.33	0.33
Gain (v)	0.0015	0.0015
Transformer Rated Capacity/MVA	1207.46	1183.62

**Table 2:** Parameters of AC filters.

Item (Unit)	Rectifier-Side Parameters			Inverter-Side Parameter		
	re 1	re 2	re 3	in 1	in 2	in 3
R	32.64	287.29	91.41	14.44	127.05	40.42
C	68.04	61.23	3.06	153.15	13.77	6.890
L	0.1496	0.0149	-	0.0661	0.0066	-

**Table 3:** Values of TOV when monopolar is blocked.

SCR	*TOV * (pu)
3	0.175
4	0.127
5	0.101
6	0.087
7	0.073
8	0.065

**Table 4:** Values of TOV when bipolar is blocked.

SCR	*TOV * (pu)
3	0.325
4	0.241
5	0.19
6	0.156
7	0.129
8	0.114

## Data Availability

All data used are available in the text.

## Supplementary Materials

The following are available online at https://www.mdpi.com/article/10.3390/s22072793/s1, Figure S1: The steady state simulation results on rectifier side, Figure S2: The steady state simulation results on inverter side, Figure S3: The transient state simulation results on rectifier side, Figure S4: the transient state simulation results on inverter side, Figure S5: Transient state performance of rectifier side under single phase to ground fault, Figure S6 Transient state performance of inverter side under single phase to ground fault, Figure S7: Transient state performance of rectifier side under three phase fault to ground, Figure S8: Transient state performance of inverter side under three phase fault to ground, Figure S9: Transient state performance of single phase to ground with severe AC fault.
