# Supplementary material for: Modeling of Efficient Control Strategies for LCC-HVDC Systems: A Case Study of Matiari–Lahore HVDC Power Transmission Line
Source: Sensors (Basel). 2022 Apr 6;22(7):2793. doi: 10.3390/s22072793 (PMC9003459; doi:10.3390/s22072793)
Supplement: Supplementary file 1 [file sensors-22-02793-s001.zip › sensors-1519919-supplementary.pdf]

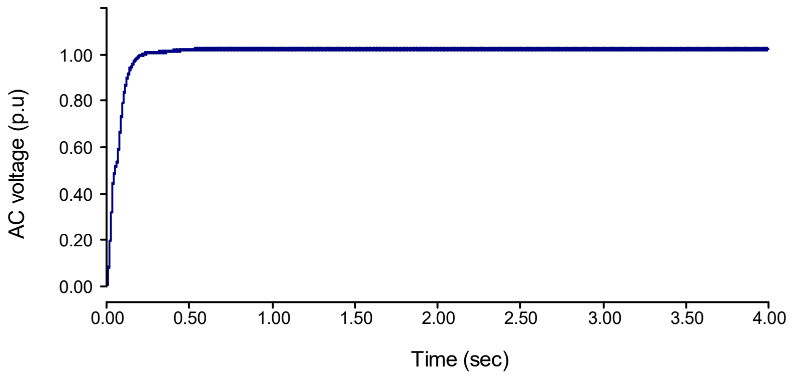

(a) AC voltage

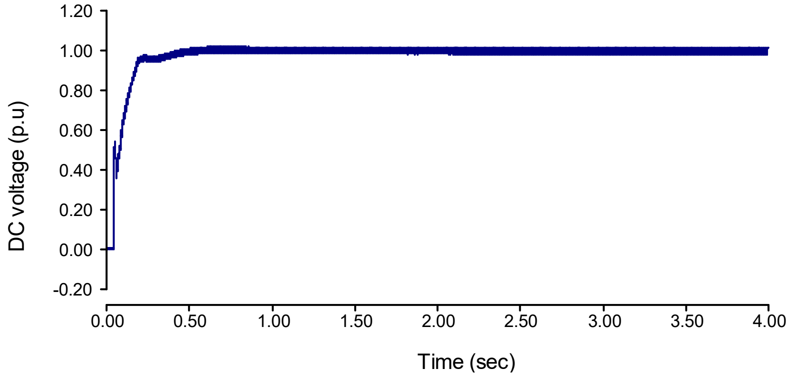

(b) DC voltage

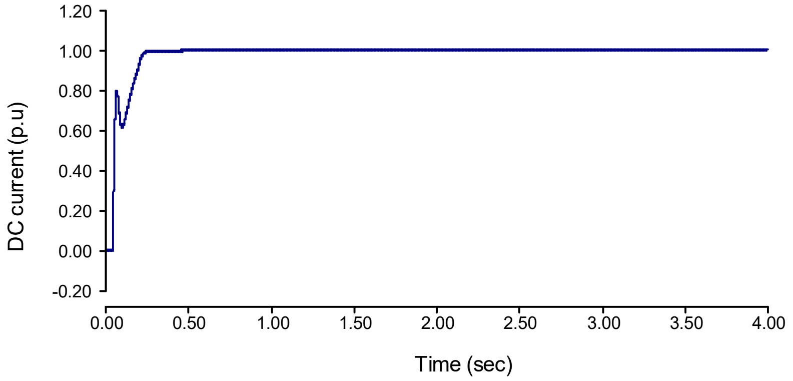

(c) DC current

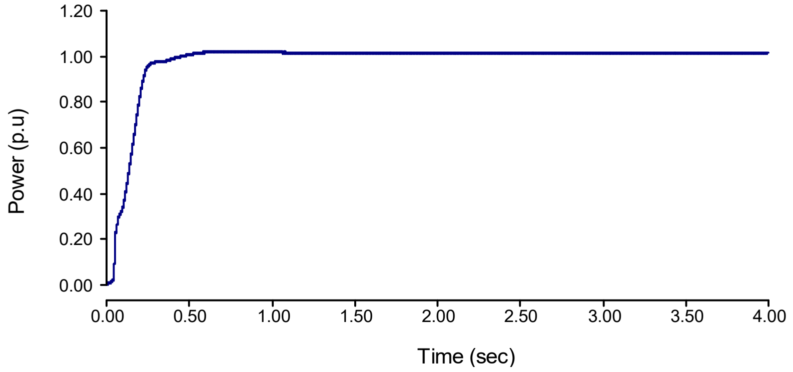

(d) Active power

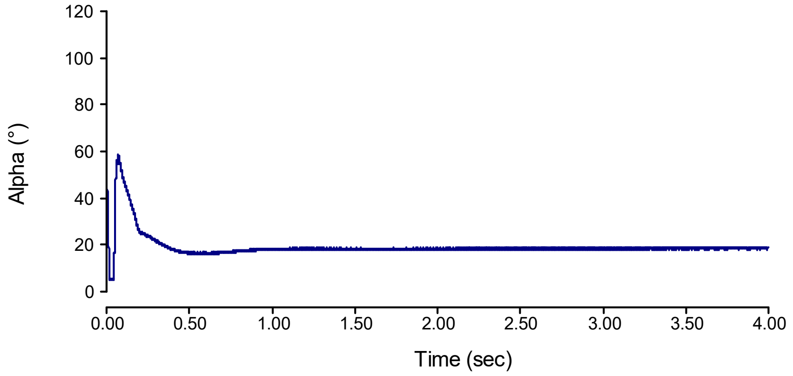

(e) α

Figure S1 The steady state simulation results on rectifier side

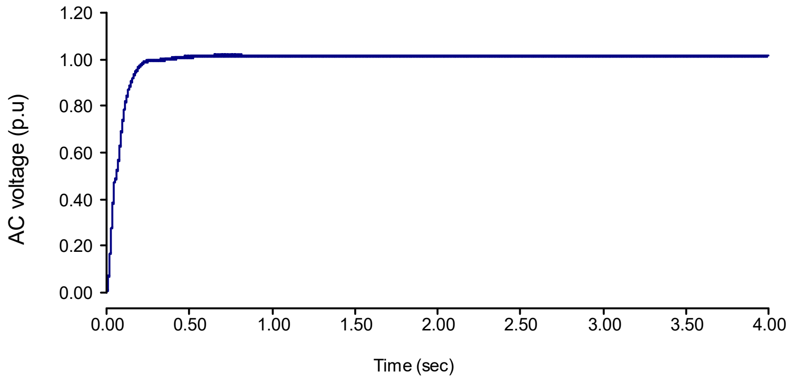

(a) AC voltage

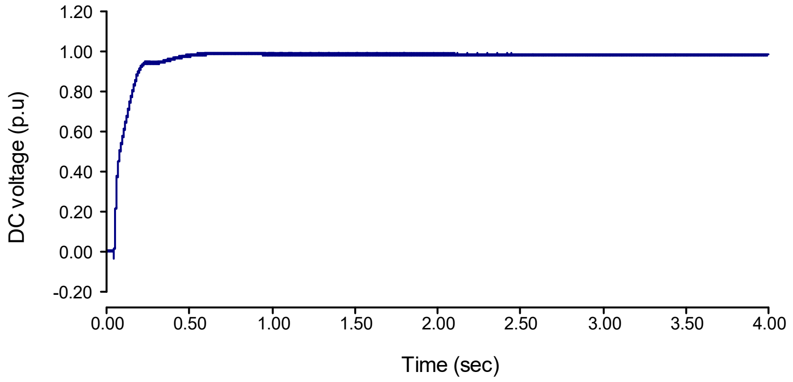

(b) DC voltage

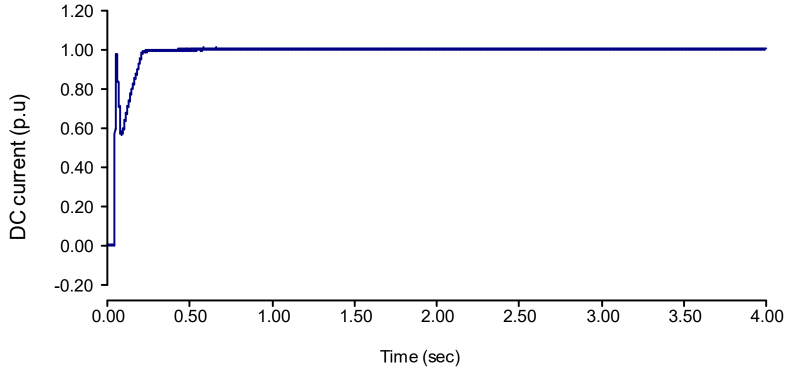

(c) DC current

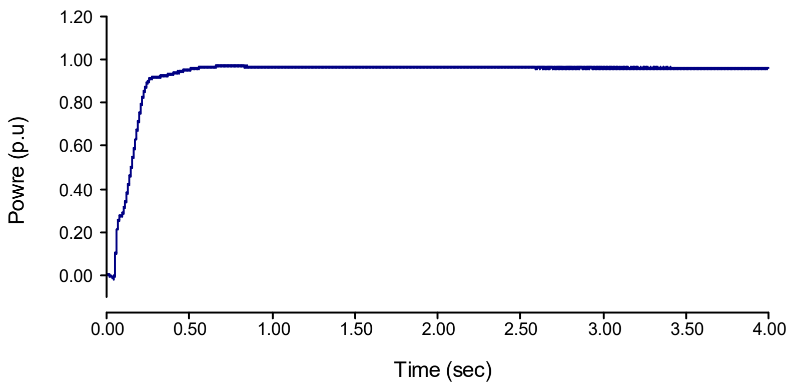

(d) Active power

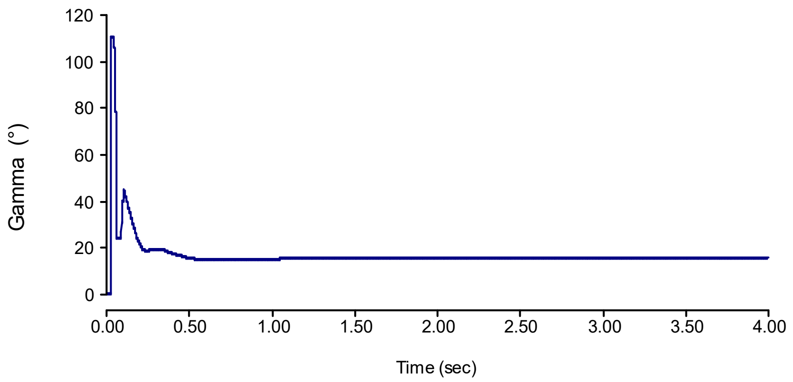

(e) γ

Figure S2 The steady state simulation results on inverter side

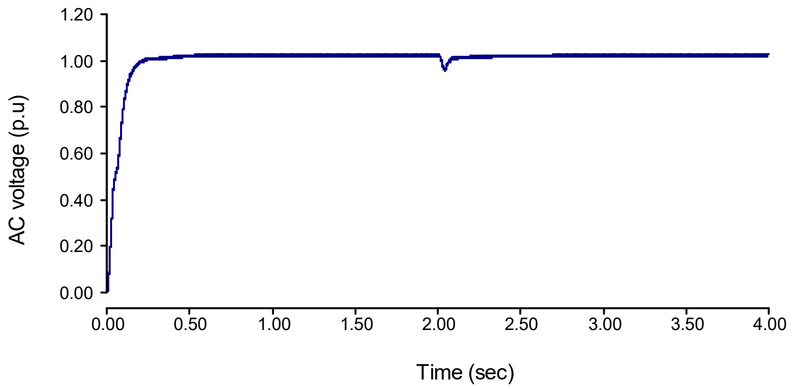

(a) AC Voltage

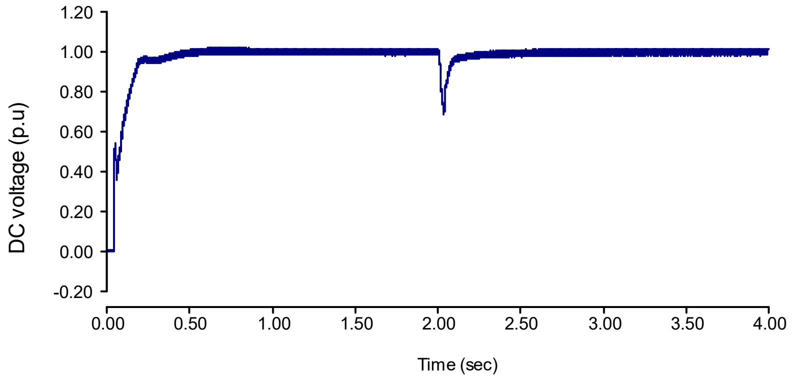

(b) DC voltage

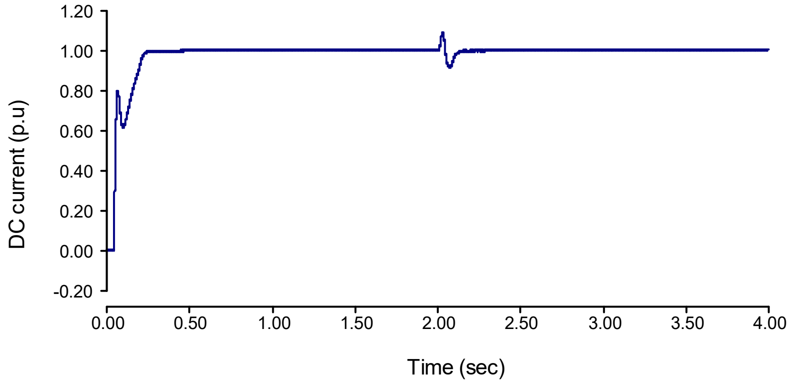

(c) DC current

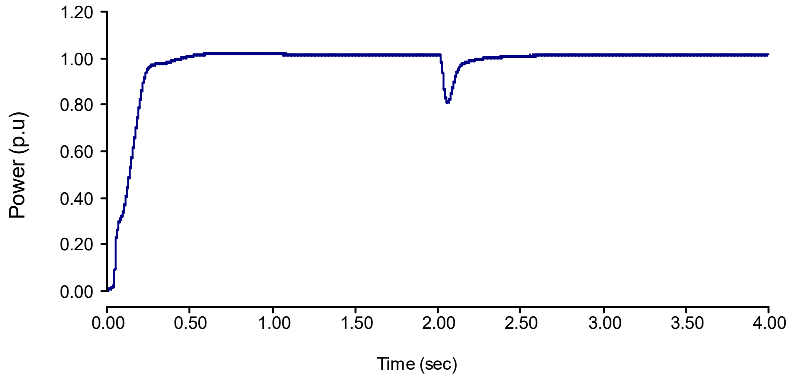

(d) AC power

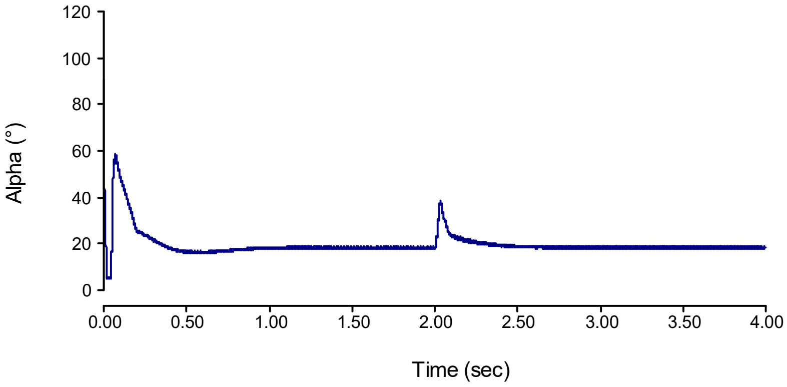

(f) Alpha (α)

Figure S3 The transient state simulation results on rectifier side

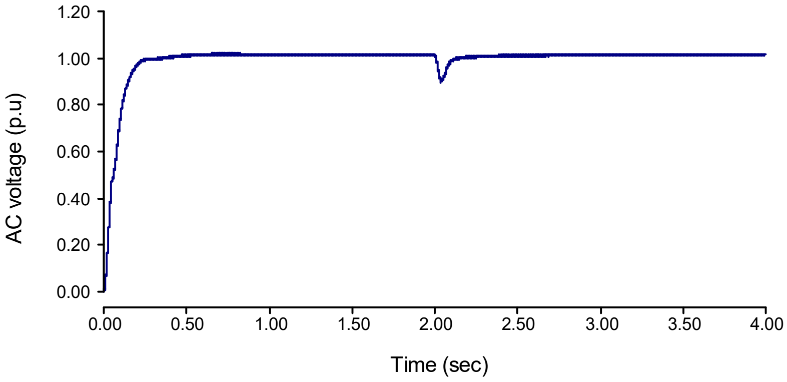

(a) AC voltage

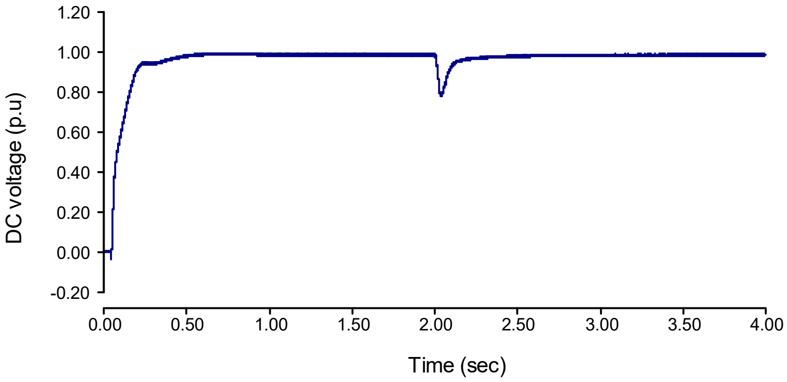

(b) DC voltage

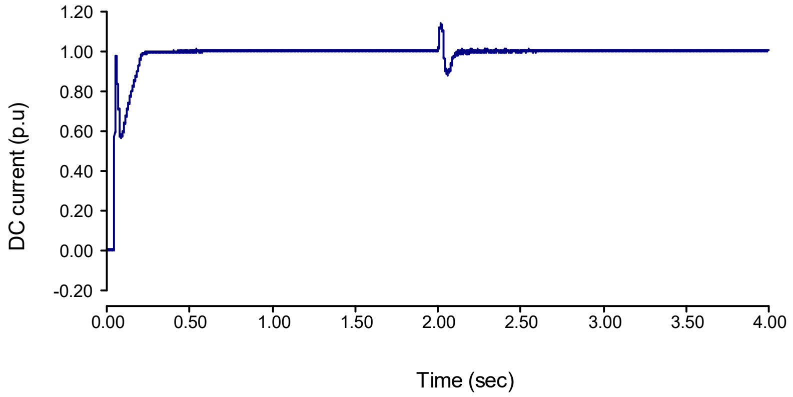

(c) DC current

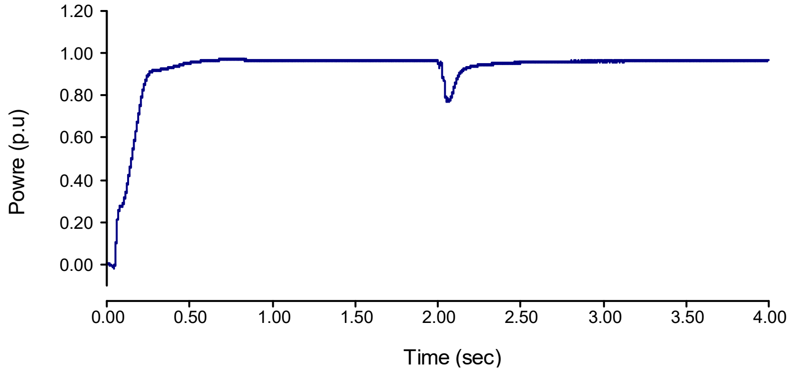

(d) AC power

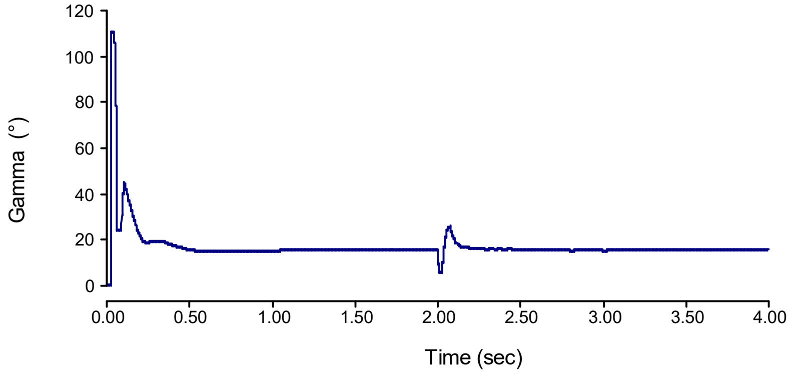

(e) γ

Figure. S4 the transient state simulation results on inverter side

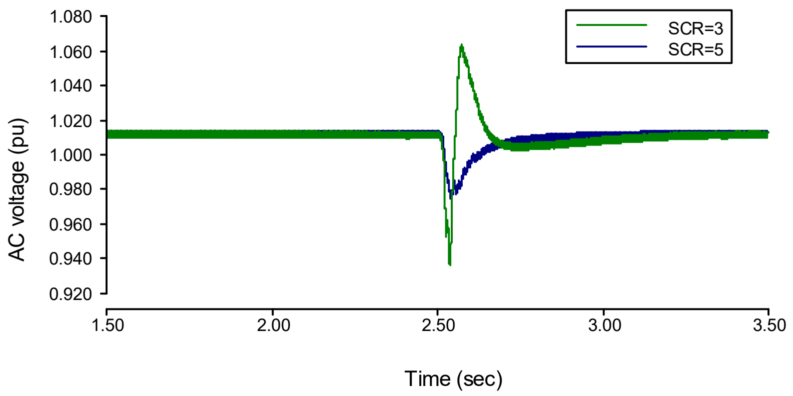

(a) AC Voltage

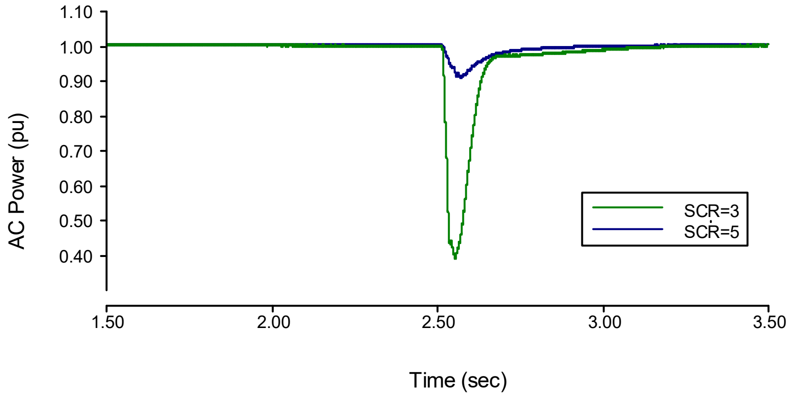

(b) AC Power

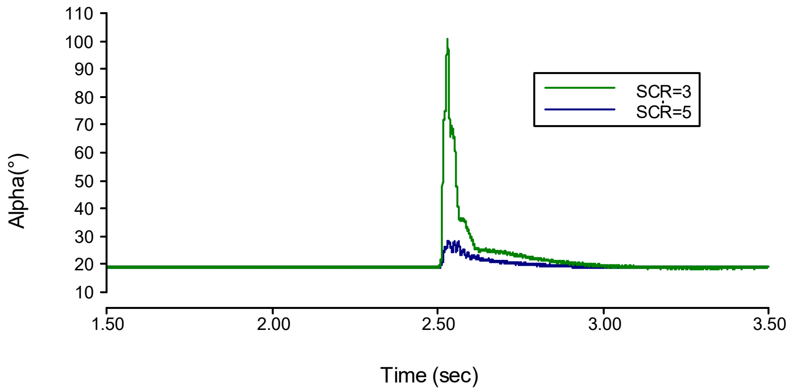

(c) Alpha (α)

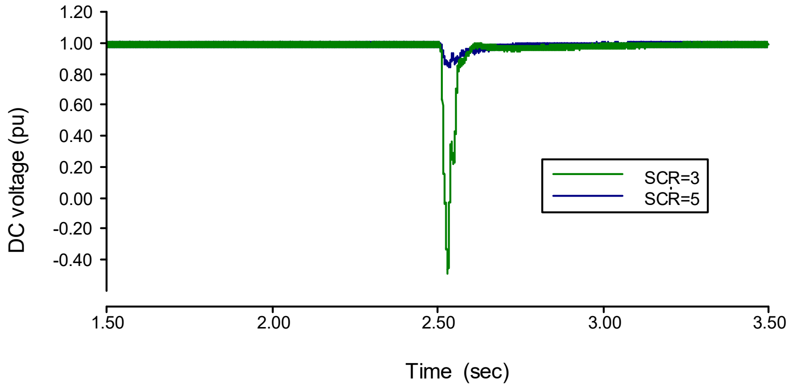

(d) DC Voltage

Figure S5 Transient state performance of rectifier side under single phase to ground fault

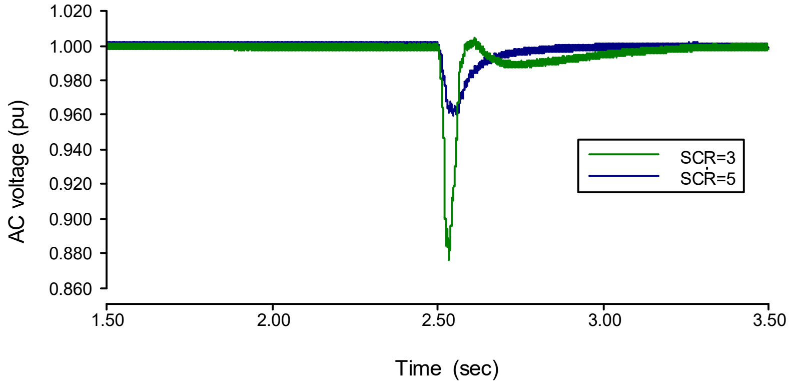

(a) AC voltage

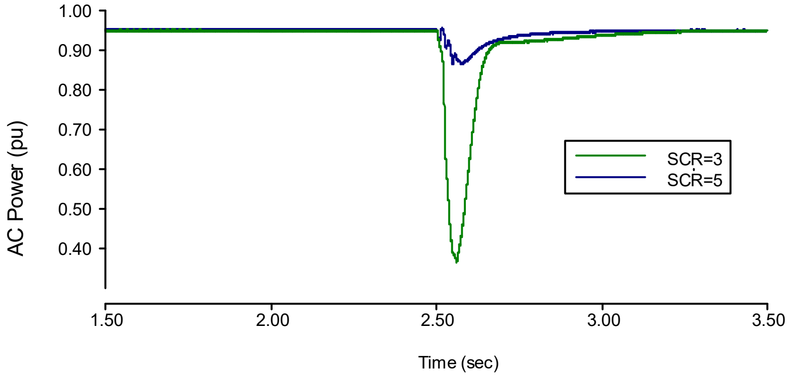

(b) AC Power

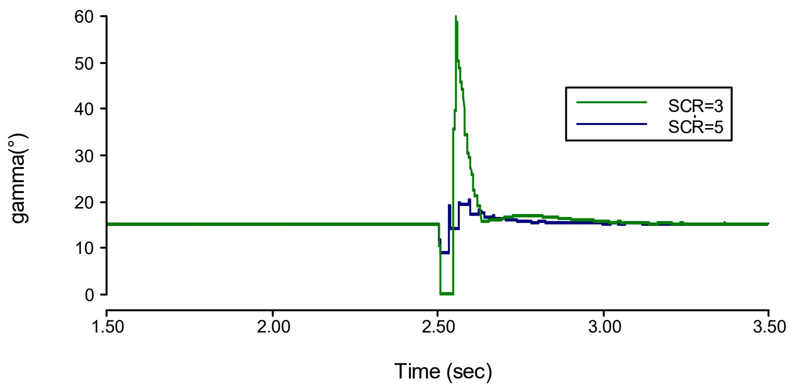

(c) γ

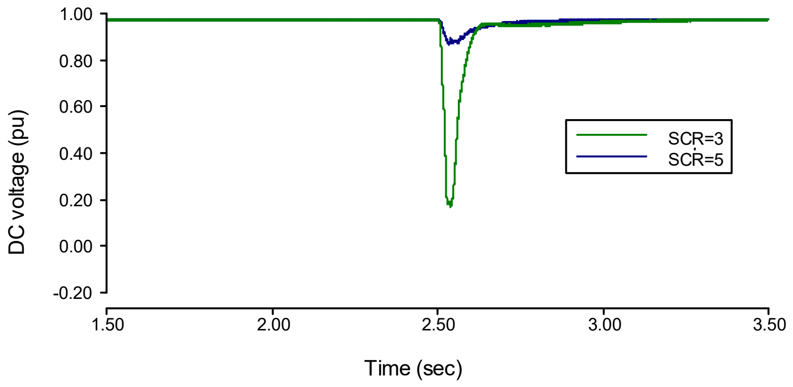

(d) DC Voltage

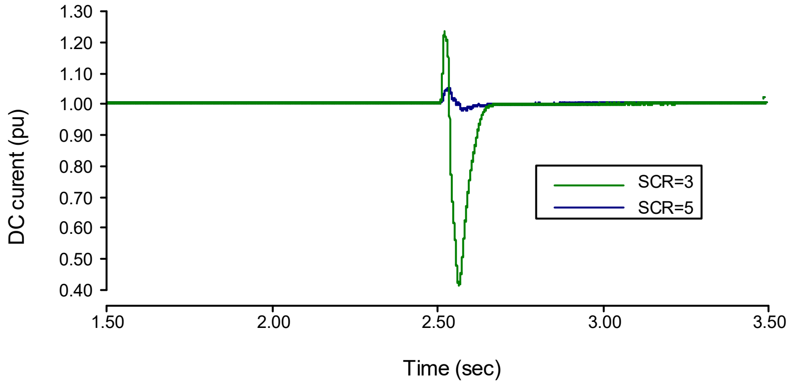

(e) DC Current

Figure S7 Transient state performance of inverter side under single phase to ground fault

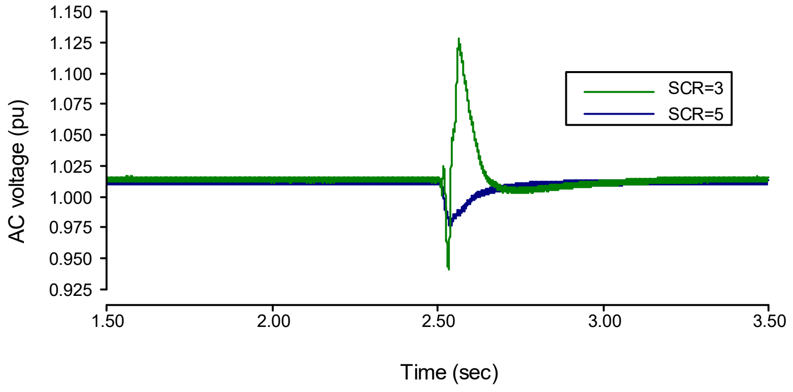

(a) AC Voltage

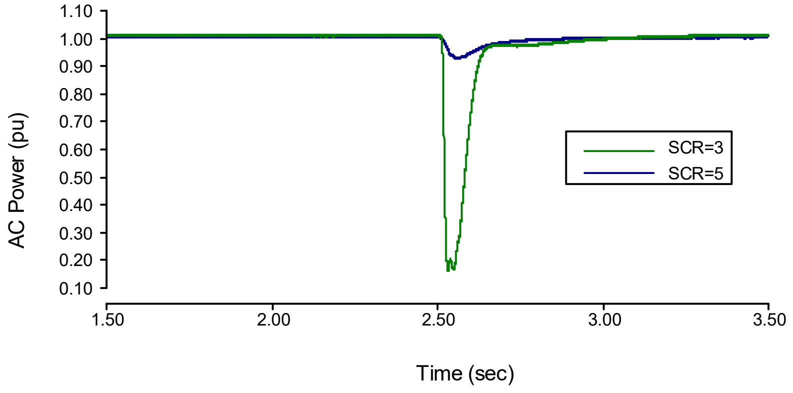

(b) AC Power

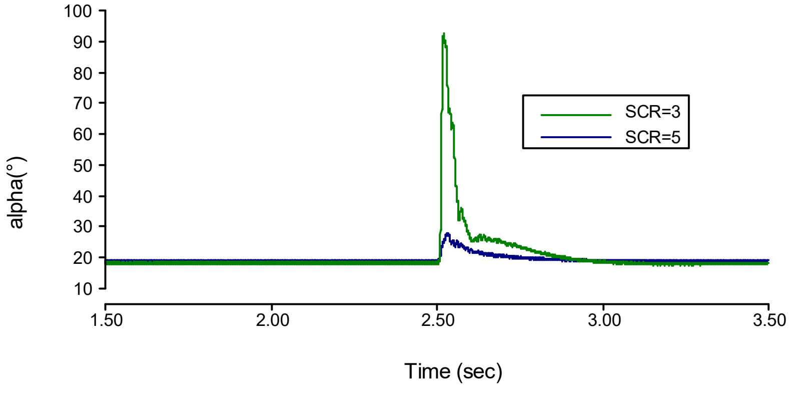

(c)Alpha (α)

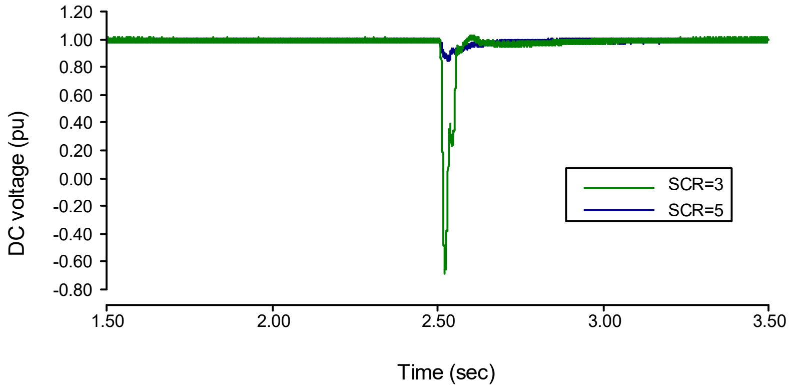

(d) DC voltage

Figure S8 Transient state performance of rectifier side under three phase fault to ground

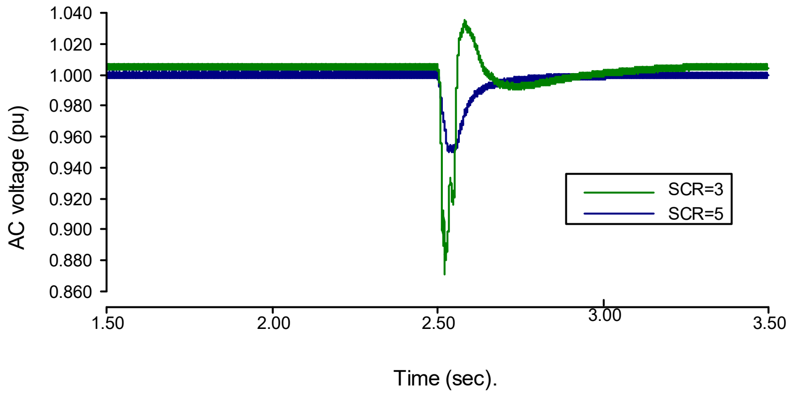

(a) AC voltage

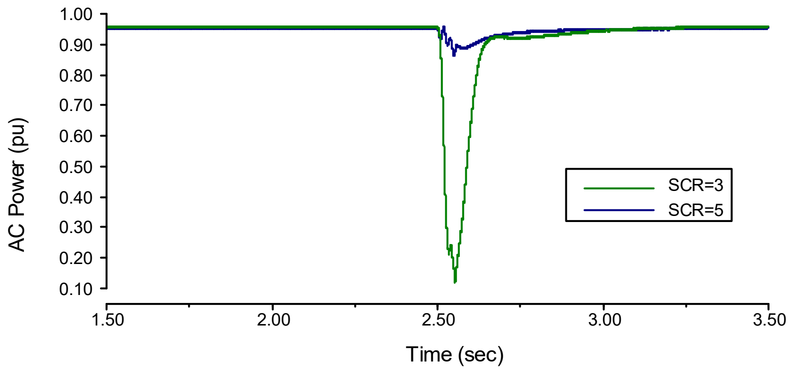

(b) AC Power

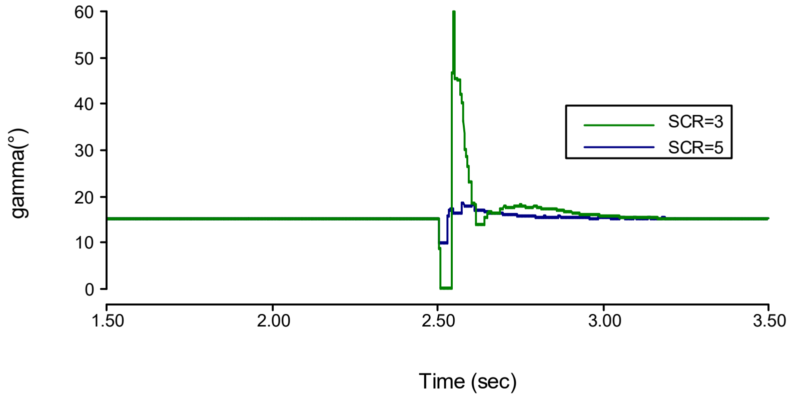

(c) γ

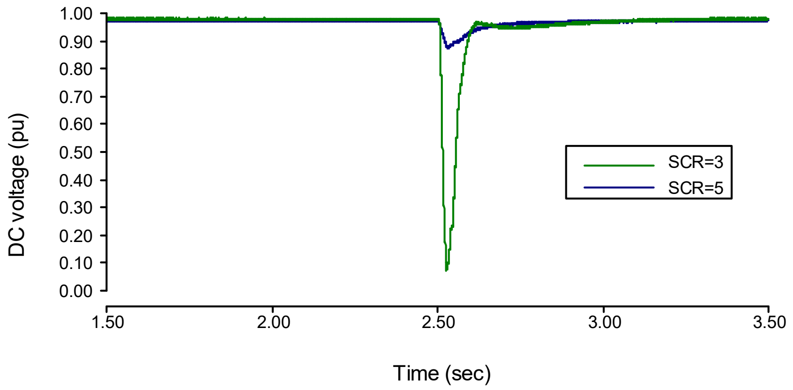

(d) DC Voltage

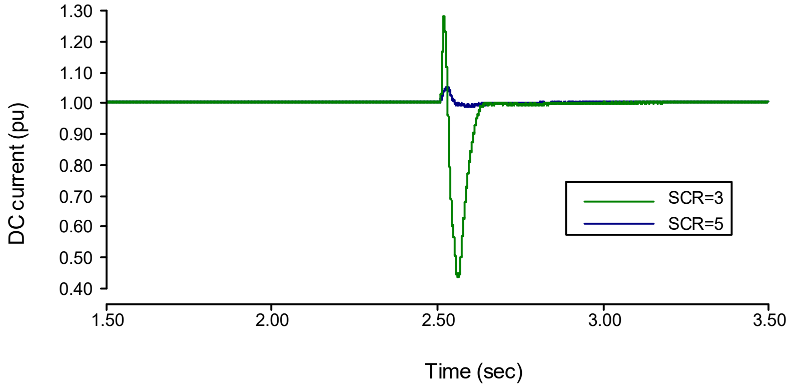

(e) DC Current

Figure S8 Transient state performance of inverter side under three phase fault to ground

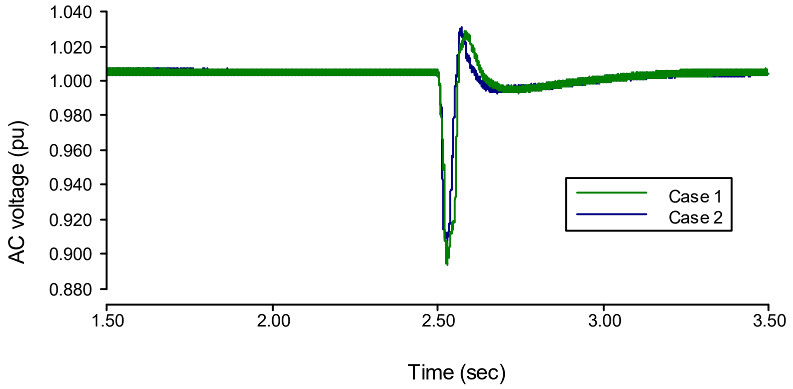

(a) AC voltage

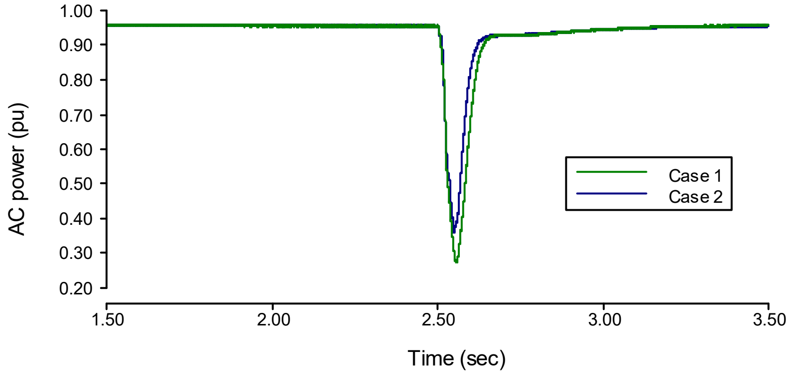

(b) AC power

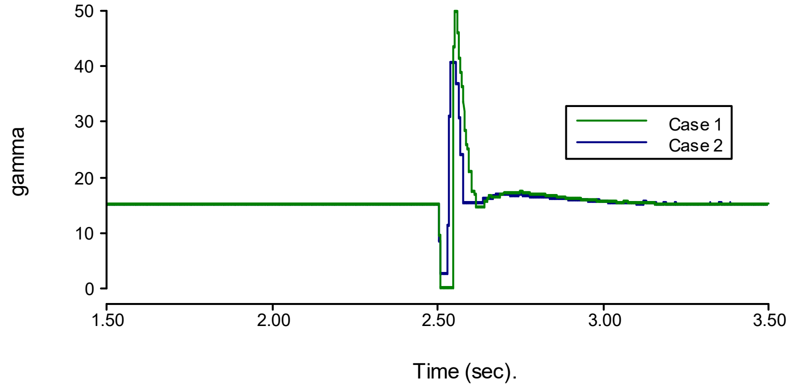

(c) gamma

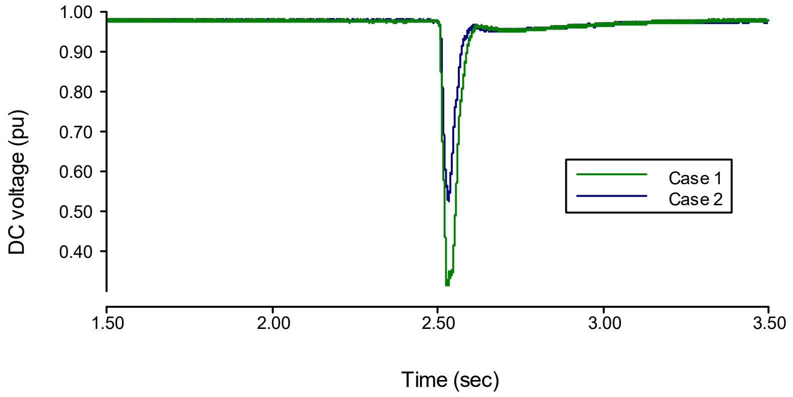

(d) DC voltage

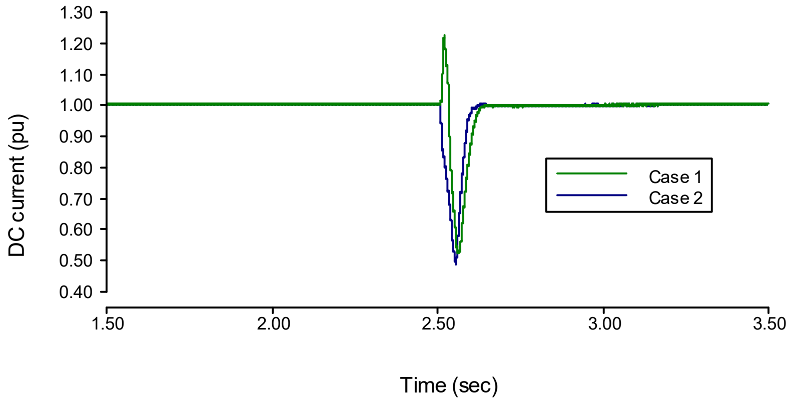

(e) Current order

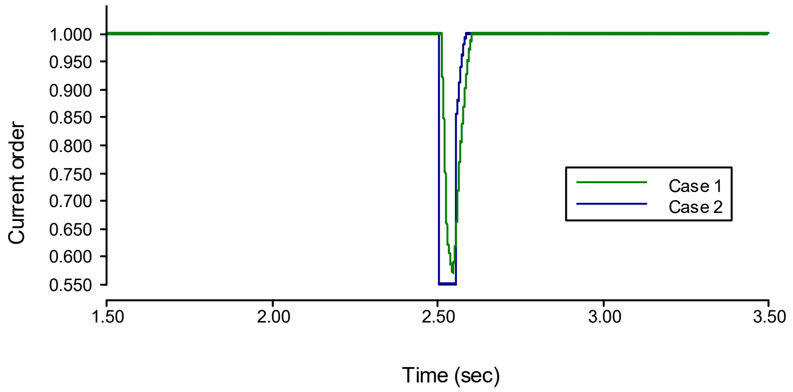

(f) DC current order

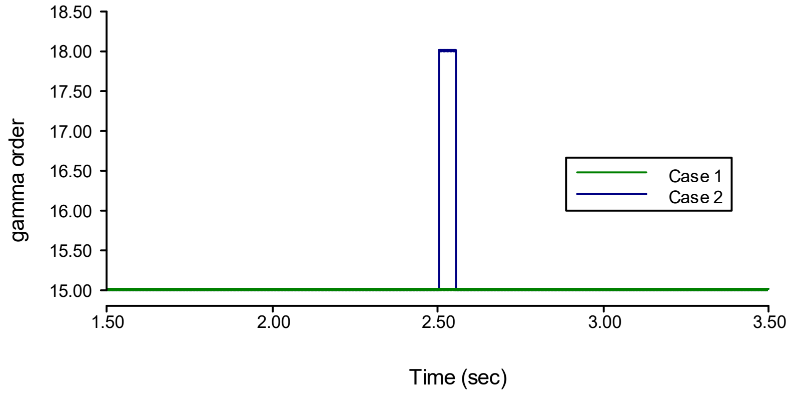

(g) gamma order

Figure S 9 Transient state performance of single phase to ground with severe AC fault
